# The Mississippi Delta Health Collaborative Medication Therapy Management Model: Public Health and Pharmacy Working Together to Improve Population Health in the Mississippi Delta

**DOI:** 10.5888/pcd17.200063

**Published:** 2020-09-17

**Authors:** Leigh Ann Ross, Lauren S. Bloodworth, Meagan A. Brown, Scott S. Malinowski, Rebecca Crane, Victor Sutton, Masoumeh Karimi, A. Cassandra Dove Brown, Thomas Dobbs, Lisle Hites

**Affiliations:** 1 University of Mississippi School of Pharmacy, Oxford, Mississippi; 2 Aaron E. Henry Community Health Services Center, Clarksdale, Mississippi; 3 Mississippi State Department of Health, Jackson, Mississippi; 4 University of Alabama, Tuscaloosa, Alabama

## Abstract

**Introduction:**

The Mississippi Delta has high rates of chronic disease and is known for its poor health outcomes and health disparities. The University of Mississippi School of Pharmacy (UMSOP) and the Mississippi State Department of Health partnered in 2009 through the Mississippi Delta Health Collaborative to reduce health disparities and improve clinical outcomes by expanding the UMSOP’s evidence-based medication therapy management (MTM) initiative, focused in Mississippi’s 18-county Delta region, to federally qualified health centers (FQHCs) in 4 of those counties.

**Methods:**

Between January 2009 and August 2018, the MTM initiative targeted FQHC patients aged 18 years or older with a diagnosis of diabetes, hypertension, and/or dyslipidemia. Pharmacists initially met face-to-face with patients to review all medications, provide education about chronic diseases, identify and resolve drug therapy problems, and take appropriate actions to help improve the effectiveness of medication therapies. Clinical parameters evaluated were systolic blood pressure (SBP), diastolic blood pressure (DBP), total cholesterol, low-density lipoprotein (LDL) cholesterol, triglycerides, and hemoglobin A_1c_ (HbA_1c_).

**Results:**

The analysis included 335 patients with hypertension (n = 287), dyslipidemia (n = 131), and/or diabetes (n = 331). Significant mean reductions occurred in the following metrics: SBP (7.1 mm Hg), DBP (6.3 mm Hg), LDL cholesterol (24.9 mg/dL), triglycerides (45.5 mg/dL), total cholesterol (37.7 mg/dL), and HbA_1c_ (1.6% [baseline ≥6%] and 1.9% [baseline ≥9%]).

**Conclusion:**

Despite the cultural and environmental disadvantages present in the Mississippi Delta, the integrated MTM treatment program demonstrated significant health improvements across 3 chronic diseases: hypertension, dyslipidemia, and diabetes. This model demonstrates that a partnership between public health and pharmacy is a successful and innovative approach to care.

**Summary**

**What is known on this topic?**

The Mississippi Delta has high rates of chronic disease and is known for its poor health outcomes and health disparities. Medication therapy management (MTM) improves the safe and effective use of medications, and ensuring appropriate medication use can improve clinical outcomes related to cardiovascular disease (CVD).

**What does this research add to the literature?**

Pharmacists met face-to-face in federally qualified health centers with patients who had a diagnosis of diabetes, hypertension, and/or dyslipidemia to provide MTM. Patients experienced mean reductions in systolic and diastolic blood pressure, low-density lipoprotein cholesterol, triglycerides, total cholesterol, and hemoglobin A_1c_.

**What are the implications for public health practice?**

MTM is an effective way to improve CVD outcomes in residents of regions like the Mississippi Delta that have high rates of poverty, health disparities, and poor health outcomes.

## Introduction

The 18 counties of the Mississippi Delta are characterized by high levels of poverty, high prevalence of chronic disease, and mortality rates that significantly exceed the national average (1,2). Moreover, regional mortality rates have increased during the past 4 decades, even as national rates have decreased (2). As of 2017, the cardiovascular disease (CVD)-attributed mortality rate was the highest in the nation, and rates have continued to increase (3,4). The difficulties experienced in the Mississippi Delta are further exacerbated by disparities related to sex and race/ethnicity (5).

Medications are an important aspect of the treatment of chronic disease; 5.8 billion prescriptions were filled in the United States in 2018 (6). Medication therapy management (MTM) improves the safe and effective use of medications, including resolving drug therapy problems, promoting adherence, and increasing continuity of care, as well as improving measures of patient and provider satisfaction (7–13). Ensuring appropriate medication use can improve CVD clinical outcomes, reduce mortality rates, and decrease health care costs (14). 

To address the detrimental effect of CVD in this region, the University of Mississippi School of Pharmacy (UMSOP) started a community-based research program in 2008 that implemented pharmacist-delivered MTM services. That same year, the Mississippi State Department of Health, with funding from the Centers for Disease Control and Prevention, created the Mississippi Delta Health Collaborative (MDHC) to implement evidence-based strategies in the Mississippi Delta for CVD prevention and management. With this shared goal of improving cardiovascular outcomes for patients in this region, the UMSOP and the Mississippi State Department of Health partnered in 2009 to expand the MTM initiative from community pharmacies into federally qualified health centers (FQHCs) in 4 Mississippi Delta counties where CVD and health disparities were prevalent and MTM services were not readily available.

## Methods

The UMSOP implemented a program to integrate pharmacists as members of health care teams at FQHCs and provide MTM services focused on CVD risk reduction in underserved patients in rural Mississippi. MTM services were provided and evaluated in 4 FQHCs in the Mississippi Delta: Aaron E. Henry Community Health Services Center in Batesville (Panola County) and Clarksdale (Coahoma County), G.A. Carmichael Family Health Center in Yazoo City (Yazoo County), and Vicksburg-Warren Family Health Care Clinic (Warren County). Between January 2009 and August 2018, the MTM initiative enrolled FQHC patients aged 18 years or older with a diagnosis of diabetes, hypertension, and/or dyslipidemia. Patients were included in the outcomes analysis if they had at least 1 follow-up visit within 12 months after enrollment. This project was approved by the University of Mississippi institutional review board.

For the clinical outcomes analysis portion of this partnership evaluation, we focused on the most recent 12-month period funding cycle. This period was chosen because it was most representative of the culmination of our partnership efforts and clinical practice guidelines.

**Intervention.** Participating patients were current FQHC patients referred to the program by practitioners of participating clinics in an attempt to improve outcomes of existing chronic diseases they were being treated for, patients newly diagnosed with 1 of the identified chronic diseases, or patients at risk for CVD. Services provided were developed based on the MTM Core Elements Service Model, which includes medication therapy review, personal medication record, medication-related action plan, intervention or referral, and documentation and follow-up (15). Upon consent and enrollment, clinical pharmacists set appointments to see patients for an initial encounter. Before the face-to-face encounter, the pharmacists reviewed patients’ records to determine what measures were needed to help patients achieve their desired health goals.

**Initial pharmacist MTM visit**. During the 60-minute initial visit, the pharmacist performed any number of the following activities depending on the patient’s needs, including but not limited to the following:

- Conducting a comprehensive medication review and a medication reconciliation
- Identifying and resolving potential and actual drug therapy problems
- Assessing clinical parameters, including systolic blood pressure (SBP), diastolic blood pressure (DBP), total cholesterol, low-density lipoprotein (LDL) cholesterol, triglycerides, and hemoglobin A_1c_ (HbA_1c_) to determine needed changes in therapy or other intervention
- Taking any appropriate actions to help improve the effectiveness of medication therapies, including initiating or modifying medication therapy via collaborative practice agreement or through recommendations to the primary provider
- Developing a medication action plan, which may include changes to medication therapy
- Delivering health education on chronic disease state and self-management practices
- Providing patient with medication adherence tools
- Initiating laboratory monitoring (including noninvasive monitoring, such as self-monitoring blood pressure or blood glucose)
- Communicating medication therapy changes and recommendations with primary provider via electronic health record or other mechanism
- Facilitating any additional referrals (eg, primary care provider, specialist providers, community health worker, social work, podiatry, optometry)

**Follow-up pharmacist MTM visit**. At the conclusion of the initial visit, the pharmacist scheduled a follow-up visit to help the patient monitor health conditions, review medication therapies, provide any additional education or counseling needed, and take appropriate actions to more effectively manage health conditions. Pertinent activities from the initial visit may occur on the follow-up visit for the pharmacist assessment, intervention, and plan. Following each visit, the pharmacist documented the encounter details in the patient’s electronic health record to share and communicate MTM recommendations with members of the clinical team and update patient action plans and medication profiles.

**Medically relevant tests.** Blood pressure was measured and evaluated at each visit with the pharmacist. If a diagnosis of diabetes was present, HbA_1c_ levels were checked at the initial visit and then every 3 months or as deemed appropriate. A lipid panel was obtained at the initial visit and then every 3 months or as deemed appropriate.

Clinical data were collected throughout MTM implementation. Clinical parameters evaluated were SBP, DBP, LDL cholesterol, triglycerides, total cholesterol, and HbA_1c_. Clinical data were dependent on clinical diagnoses, specifically hypertension, dyslipidemia, diabetes, or any combination of the 3. Accordingly, HbA_1c_ percentage was collected for patients with a diagnosis of diabetes. LDL cholesterol, triglycerides, and total cholesterol were collected for patients with dyslipidemia. SBP and DBP were collected for all patients; however, only those presenting with elevated levels were included in analyses. Furthermore, there was considerable overlap or comorbidity in patient diagnoses; therefore, totals for the reported number of participants were not mutually exclusive from those of other chronic disease conditions. Because there was no expectation of improvement for patients with clinical measures that were normal at baseline, these patients’ data for those variables were not included in the analysis.

For comparative purposes, patient data were analyzed by duration of participation in the MTM initiative. Time zero (T1), the pre-MTM intervention measure, was compared with the post-MTM intervention measure (T2). Hypertensive patients’ T2 measures were collected 6 to 12 months into participation in the MTM initiative, and T2 measures for patients with other chronic disease diagnoses were collected 9 to 12 months into program participation. Because hypertensive patients typically experienced rapid improvement after beginning the MTM intervention and had shorter participation duration, we extended their time frame for analysis. Participation duration–based analysis was needed to aggregate participants across the many years of the initiative and to facilitate the rolling admissions process, allowing a pre–post within-factor design. We used *t* tests to assess significance of clinical change. Accordingly, any patients with fewer than 2 clinical assessments at least 6 months apart were excluded from the study. Patients who presented with elevated clinical numbers and were enrolled in the MTM program but who had their blood pressure at goal were also excluded from hypertension analyses. For patients with multiple clinical measures in the T2 point, we used a mean of those measures.

Finally, hypertension was further analyzed by stage, broken into 4 groups according to severity of elevated blood pressures, using the higher stage of SBP or DBP at the time measurement. The Figure displays the cut-off scores used to determine normal versus elevated levels for each clinical measure, as well as a breakdown of hypertension stages used in this analysis.

**Figure:**
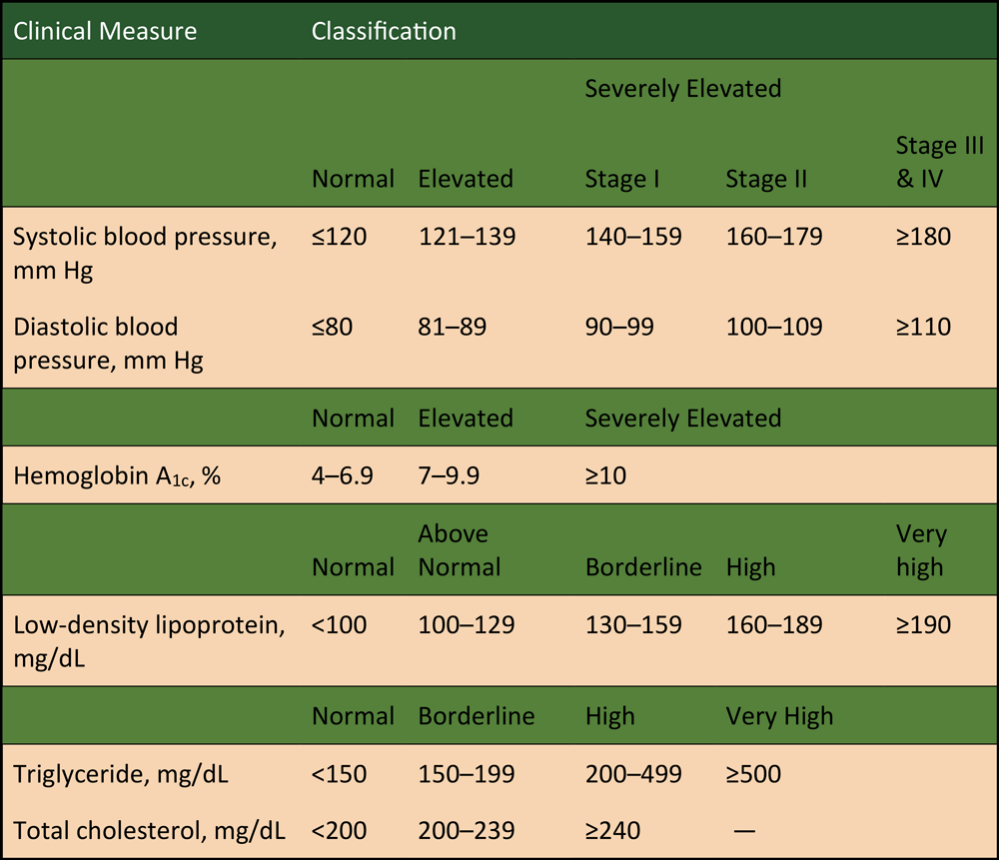
Classification of disease states, by severity, Mississippi Delta Health Collaborative Medication Therapy Management Model, 2009–2018. Hypertension staging was based on clinical guidelines from the 8th Joint National Committee for the Management of Hypertension in Adults (31). Abbreviation: —, not applicable.

**Table:** 

Clinical Measure	Normal	Elevated	Severely Elevated		
			Stage I	Stage II	Stage III and IV
Systolic blood pressure, mm Hg^a^	≤120	121–139	140–159	160–179	≥180
Diastolic blood pressure, mm Hg^a^	≤80	81–89	90–99	100–109	≥110
Hemoglobin A1c (%)	4–6.9	7–9.9	10+		
	**Normal**	**Above Normal**	**Borderline**	**High**	**Very High**
Low-density lipoprotein, mg/dL	<100	100–129	130–159	160–189	≥190
	**Normal**	**Borderline**	**High**	**Very High**	
Triglyceride, mg/dL	<150	150–199	200–499	≥500	
Total cholesterol, mg/dL	<200	200–239	≥240	—	

## Results

A total of 335 patients met the inclusion criteria for analysis. This represented a 71.3% retention rate (ie, 335 of 470 patients returned for a follow-up visit within 1 year after enrollment and were included in the comparative analysis). This population averaged 2.4 total visits per year. Grouped by diagnosis, 287 patients with hypertension, 131 patients with dyslipidemia, and 331 patients with diabetes were included in the analyses. Patients were 61.2% female and had a mean age of 60 years. The population studied was 95.0% Black, 4.5% White, and 0.5% other race.

MTM participant outcome data (Table 1) include mean baseline or pre-MTM intervention mean scores (T1) and mean post-MTM intervention scores (T2), as well as actual and relative change in each clinical measure. All clinically relevant metrics demonstrated significant improvement (*P* < .01; range, –4.2% for SBP to –18.2% for triglycerides). 

**Table 1:** Overall MTM Clinical Laboratory Outcomes Within the First Year of Enrollment, Mississippi Delta Health Collaborative, 2009–2018^a^

Clinical Measure	No.	Baseline Mean	Post-MTM Mean	Change	*P* Value^b^	Relative % Change
Systolic blood pressure, mm Hg	287	142.7	135.6	–7.1	<.001	–4.2
Diastolic blood pressure, mm Hg	191	89.9	83.6	–6.3	<.001	–7.0
LDL cholesterol, mg/dL	112	140.9	116.0	–24.9	<.001	–17.6
Triglycerides, mg/dL	70	249.9	204.4	–45.5	.001	–18.2
Total cholesterol, mg/dL	82	245.3	207.6	–37.7	<.001	–15.3
Hemoglobin A_1c_, % (Baseline ≥6)	331	10.7	9.1	–1.6	<.001	–14.8
Hemoglobin A_1c_, % (Baseline ≥9)	275	11.2	9.3	–1.9	<.001	–17.1

Abbreviation: LDL, low-density lipoprotein; MTM, medication therapy management.

a For all patients. Normal values at baseline were excluded.

b *P* values determined by using paired *t* test.

Blood pressure outcomes varied considerably across disease severity or hypertension stage (Table 2). Patients with Stage III and IV hypertension (blood pressure at or above 180 systolic and/or 110 diastolic) experienced the greatest level of improvement (16.8% and 12.5%, *P* = .002 and *P* = .01, respectively). Significant results were also experienced by Stage II (*P* < .001) and Stage I participants (*P* = .003).

**Table 2:** Blood Pressure Change Among MTM Patients (N = 298), by Hypertension Stage, Mississippi Delta Health Collaborative, 2009–2018^a^

Baseline	No. of Patients Showing Decrease in BP, No. (%)^b^	Change in BP Between Baseline and Follow-Up					
		BP type	Baseline Mean, mm Hg	Post-MTM Mean, mm Hg	Change, mm Hg	*P* Value^c^	Relative % Reduction
At Risk (n = 137)	93 (68)	Systolic	128.9	129.0	0.1	.92	—
		Diastolic	79.3	77.5	–1.8	.03	2.3
Stage I (n = 100)	77 (77)	Systolic	145.1	139.3	–5.7	.003	3.9
		Diastolic	84.9	80.9	–4.0	<.001	4.7
Stage II (n = 46)	41 (89)	Systolic	160.9	147.5	–13.4	<.001	8.3
		Diastolic	92.2	85.1	–7.1	<.001	7.7
Stage III and IV (n = 14)	14 (100)	Systolic	177.5	147.6	–29.8	.002	16.8
		Diastolic	104.4	91.3	–13.1	.01	12.5

Abbreviations: —, not applicable; BP, blood pressure; MTM, medication therapy management.

a Normal values at baseline were excluded. Second laboratory result was 6 to 9 months after first visit.

b Decrease in either systolic or diastolic blood pressure.

c *P* values determined by using paired *t* test.

## Discussion

The results of the MDHC MTM efforts strongly support the use of pharmacist-delivered MTM as a part of integrated care in rural Mississippi. MTM care delivery models have a considerable literature base to support its usefulness, although little research has targeted rural, Black populations in the Deep South (9,16–20). This program targeted a largely Black population in one of the most medically underserved areas in the United States. Despite the cultural and environmental disadvantages present in this area, the integrated MTM treatment program demonstrated significant health improvements across chronic diseases, including hypertension, dyslipidemia, and diabetes. The level of impact on clinical metrics in our study is similar to other published findings.

Pharmacists have been involved in the provision of services to ensure optimal medication use for many years. This provision has evolved from Pharmaceutical Care Services or Disease Management Services terminology in the 1990s to the consensus definition of MTM adopted by the pharmacy profession in 2004, and more recently Comprehensive Medication Management (CMM), which emphasizes the team-based approach to care. Because different terms have been used historically and the components and delivery of MTM may vary, such as with Medicare Part D MTM programs, it is important to have an understanding of the robustness of this intervention. The MDHC MTM service model incorporates the MTM core elements and aligns with the Pharmacist Patient Care Process (PPCP) (15,21). In the PPCP, the pharmacist uses a patient-centered approach in collaboration with other providers on the health care team to optimize patient health and medication outcomes. This approach is accomplished by collecting the necessary information, assessing the information collected, and analyzing the clinical effects of the patient’s therapy in the context of the patient’s overall health goals to identify and prioritize problems. The pharmacist then develops and implements an individualized, evidence-based, patient-centered care plan with other providers via collaborative practice agreement or recommendations.

This initiative used several aspects of MTM that were expected to be a good match for the needs of the Mississippi Delta, including a close working relationship between pharmacists and other care provider team members at participating clinics, following the principles of interprofessional collaborative practice, and incorporating the core elements of MTM into a robust intervention. The benefits of enhancing the team-based approach have been well documented as the quadruple aim of Interprofessional Collaborative Practice, supporting the value of close collaboration and heightened interprofessional communication on reducing the cost of care while promoting provider wellbeing and improving patient outcomes (14–18,22–26). This MTM model supports the integration of pharmacists in collaborative, team-based care models in clinic settings such as this to achieve this goal.

In our study, significant reductions were demonstrated for lipemic parameters. Serum concentrations of total cholesterol, LDL cholesterol, and triglycerides improved significantly compared with baseline. Eighty-four percent of patients with dyslipidemia also had concomitant diabetes, which is a noteworthy finding because dyslipidemia is a major risk factor for CVD in patients with diabetes (27). For patients with diabetes, significant reductions in HbA_1c_ were demonstrated. Patients with the highest risk for diabetes complications (baseline HbA_1c_ >9%) experienced a 1.9% reduction in HbA_1c_ (for a 17.1% relative reduction). Similarly, SBP and DBP (analyzed separately) were significantly lower after receiving MTM services. The relative reductions observed are clinically meaningful given the various stages of hypertension at baseline. These levels of effect on clinical parameters are consistent with other published figures regarding pharmacist-delivered MTM (9,16,28–30). Improvements such as these, combined with pharmacist coaching and counseling, will likely contribute to a reduction in risk of CVD in this population. 

In addition to improved patient outcomes, the design of this program provided for continuity of care with the pharmacist during primary care provider transition periods, which occurred several times in the clinic sites. The pharmacist was consistent and present to address concerns and facilitate the delivery of historical context during these transitional periods. The face-to-face encounters were helpful in patient assessment and in ensuring that patients had a good understanding of the plan. Many of these patients had complex disease states and comorbidities and this team-based approach provided efficiency for patients, while ensuring delivery of comprehensive patient care.

### Limitations

This practice-based implementation initiative did not allow for a control group but was structured to evaluate patient outcomes in an actual care model. In this region with this disadvantaged population, challenges are often encountered in providing health care. Patients may be unable to attend clinic visits because of lack of transportation, primary provider transitions, and other financial barriers. Although pharmacists worked with patients to identify issues hindering care and attempted to incorporate social work and other resources, patients were not always successful in overcoming these challenges and continuing care. Pharmacists communicated effectively with collaborating providers through the FQHC electronic health record; however, their systems were not structured to capture the data necessary for the evaluation of services, requiring additional electronic documentation by the pharmacists. Through this grant-supported project, the pharmacist services provided were not billed or compensated. These identified challenges set the stage for future research to explore more options for pharmacist MTM delivery, such as through telehealth, and to explore additional payment options for team-based care.

In addition to the lack of a control group, small sample size was a limitation because it precluded the ability to conduct complex analyses to account for potential confounding factors. The intent was to describe a real-world care model and experience with a focus on the benefits of partnering with a state health department, and as such, the study was not designed as a large, randomized controlled trial. The small sample size also limited interpretation and extrapolation of our findings. Although the outcome variables improved significantly compared with baseline, a causal inference cannot be established, nor can it discount the fact that the results seen might have otherwise occurred naturally over time without the intervention. Despite this model being effective in this particular setting and population, it is uncertain whether the benefits would be seen in other disease states or in a more diverse population.

We were unable to account for the potential variability among the clinics included in the analysis. All clinics were FQHCs in the Mississippi Delta region that serviced a medically underserved sociodemographic. Inherent differences or variabilities were not captured or adjusted for in clinic characteristics during the study period. The intended study population was patients at high risk for cardiovascular complications from diabetes, hypertension, and dyslipidemia, which is typical of pharmacist-provided MTM and CMM services described in the literature and from our previous experiences. Unfortunately, this introduces the possibility of our results being biased toward positive findings, as patients with normal or well-controlled metrics were not included in the design or analyses. Regression toward the mean is expected in this scenario. The main reason for only including high-risk patients was that limited resources necessitated prioritizing patients with high disease burden, and subsequently, high risk for complications.

Another potential limitation was the variability in follow-up visits. Patient acuity and medical necessity largely determined individual follow-up scheduling. A large number of no-shows and reschedulings caused further variability among subjects regarding the number of encounters with MTM pharmacists. The analysis did not account for these varying levels of exposure to the intervention. Key differences may exist in the characteristics and disease severity between patients who had multiple versus few visits with the pharmacists. Furthermore, relative reductions of measured parameters were used to compare variables. Arguably, quantifying the percentage of patients achieving therapeutic goals or targets would be insightful. However, given our previous experience, relative reductions seemed more meaningful in this medically underserved population.

Lastly, the analysis did not adjust for comorbidities. Although this lack of adjustment may have made the results inherently more conservative, it does not take into account the potential impact of comorbidities across the spectrum of the findings and outcomes. Future studies focusing more on clinical outcomes and implementation science, rather than real-world partnerships, should attempt to incorporate a propensity score analysis for comorbidities or use of a tool such as the Charlson comorbidity index.

### Conclusion

Pharmacists are well equipped and positioned as medication experts to contribute in a meaningful way to team-based, collaborative care. The partnership between the UMSOP and the Mississippi State Department of Health provided an opportunity to test and demonstrate the positive impact of this intervention on markers that influence CVD, in one of the most underserved and medically challenged regions of our country. This partnership demonstrates how public health and pharmacy can align to achieve the shared goals of preventing chronic disease and improving population health through implementation of innovative strategies such as the MDHC MTM model.
